# Supplementary material for: Antifungal Activity and Mechanism of Xenocoumacin 1, a Natural Product from Xenorhabdus nematophila against Sclerotinia sclerotiorum
Source: J Fungi (Basel). 2024 Feb 26;10(3):175. doi: 10.3390/jof10030175 (PMC10970907; doi:10.3390/jof10030175)
Supplement: Supplementary file 1 [file jof-10-00175-s001.zip › jof-2835601-supplementary.pdf]

Supplementary Material

Antifungal activity and mechanism of Xenocoumacin 1, a natural product from *Xenorhabdus nematophila* against *Sclerotinia sclerotiorum*

Method of Isolation and characterization of Xcn1

The separation procedure of Xcn1 was according to the previous methods with some modifications (Mcinerney et al. 1991, Huang et al. 2005). Briefly, the cell free supernatant (100 L) was condensed to a final volume of 20 L. Then the pH of the cell free supernatant was adjusted to 3.0 with oxalic acid. After standing overnight, the pH of the supernatant was adjusted to 6.0 with NaOH. The sediment was removed by filtration and the supernatant was loaded onto an X-5 macroporous resin column (80 mm × 60 mm) at a flow rate of 2.0 mL/min. Elution with 20 L of methanol aqueous solution (50%, v/v). Subsequently, the column was eluted with acetone aqueous solution (20 L, 30%, v/v) containing 0.01M HCl. The eluate of the acetone fraction was collected and concentrated with a vacuum rotary evaporator. Then the concentrated solution was loaded onto a 110 cation exchange chromatography column. Elution with 0.2 M NH_4Cl and the eluate was desalted using an X-5 macroporous resin column. Further purification was carried out on a CM-Sephadex-C-25 cation exchange resin, and the bound fraction was eluted with 0.1 M NH_4Cl. After desalination, the active fractions containing Xcn1 were collected and lyophilized. The structure of Xcn1 was confirmed by ^{1}HNMR, ^{13}C-NMR and HR-MS. The purity was determined using a HPLC method (Agilent 1260 series HPLC system). Five microliters of sample were injected into a ZORBAX SB–C_{18} column (4.6 mm × 150 mm, 5 µm particle size), eluted with an acetonitrile-water mixture (1:9, v/v) containing 0.1% TFA at a flow rate of 0.3 mL/min, and monitored at 312 nm.

Table S1. The plant pathogenic fungi used in this study

Pathogenic fungi	Host plant	Geographic origins
Sclerotinia sclerotiorum	Oilseed rape	Hanzhong
Rhizoctonia solani	Corn Rice	Yangling
Botrytis cinerea	Tomato	Yulin
Exserohilum turcicum	Wheat	Yangling
Alternaria alternate	cucumber	Yangling
Fusarium graminearum	Wheat	Yangling
Alternaria solani	Tamato	Hanzhong
Colletotrichum gloeosporioides	Apple	Yangling
Gaeumannomyces graminis	Wheat	Yangling
Fusarium oxysporum	tobacco	Mianchi

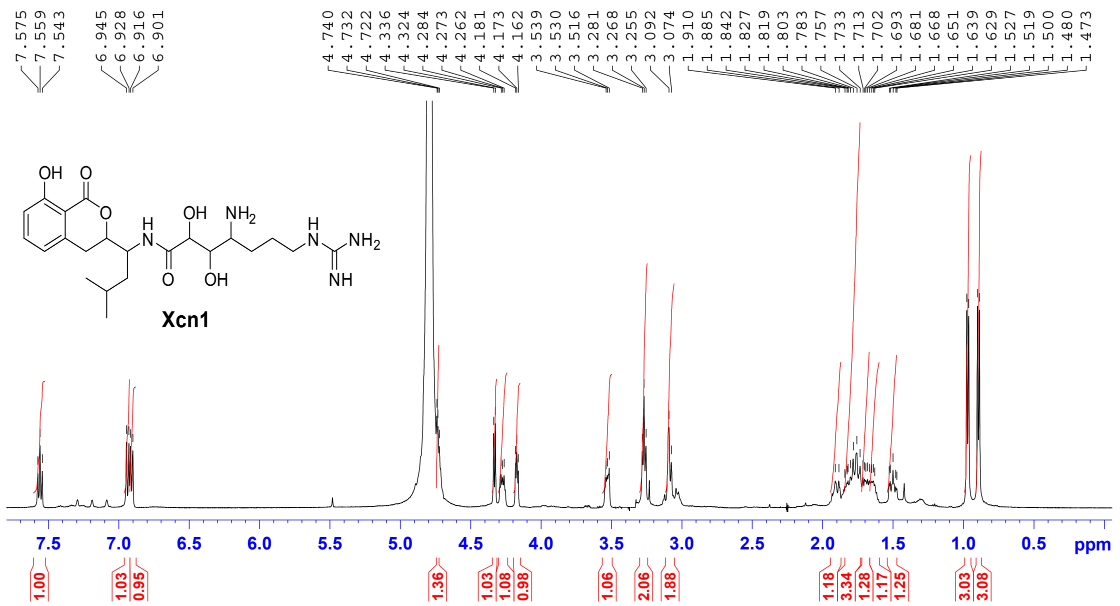

Figure S1. ^{1}H NMR spectrum of Xcn1.

^{1}H NMR (500 MHz, D_2O) δ 7.56 (t, J = 7.9 Hz, 1H), 6.94 (d, J = 8.5 Hz, 1H), 6.91 (d, J = 7.4 Hz, 1H), 4.69-4.79 (m, 1H), 4.33 (d, J = 6.0 Hz, 1H), 4.27 (dt, J = 9.8, 4.8 Hz 1H), 4.17 (dd, J = 6.0, 4.0 Hz, 1H), 3.53 (td, J = 9.8, 6.6 Hz 1H), 3.27 (t, J = 6.6 Hz, 2H), 3.07-3.10 (m, 2H), 1.95-1.87 (m, 1H), 1.85-1.74 (m, 3H), 1.73-1.66 (m, 1H), 1.66-1.59 (m, 1H), 1.50 (td, J = 9.6, 4.7 Hz, 1H), 0.97 (d, J = 6.6 Hz, 3H), 0.89 (d, J = 6.6 Hz, 3H).

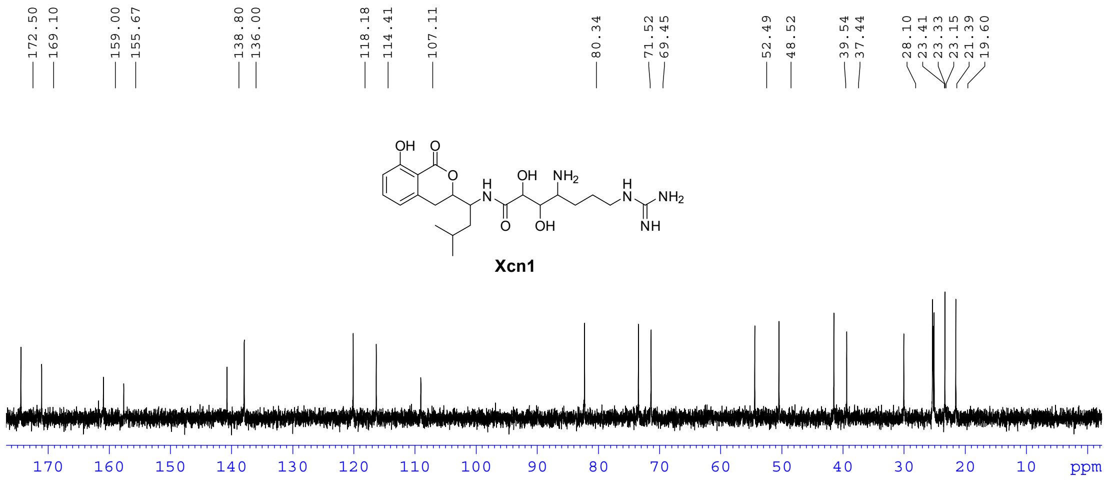

Figure S2. ^{13}C NMR spectrum of Xcn1.

^{13}C NMR (125 MHz, D_2O) δ 172.50, 169.10, 159.00, 155.67, 138.80, 136.00, 188.18, 114.41, 107.11, 80.34, 71.52, 69.45, 52.49, 48.52, 39.54, 37.44, 28.10, 23.41, 23.33, 23.15, 21.39, 19.60.

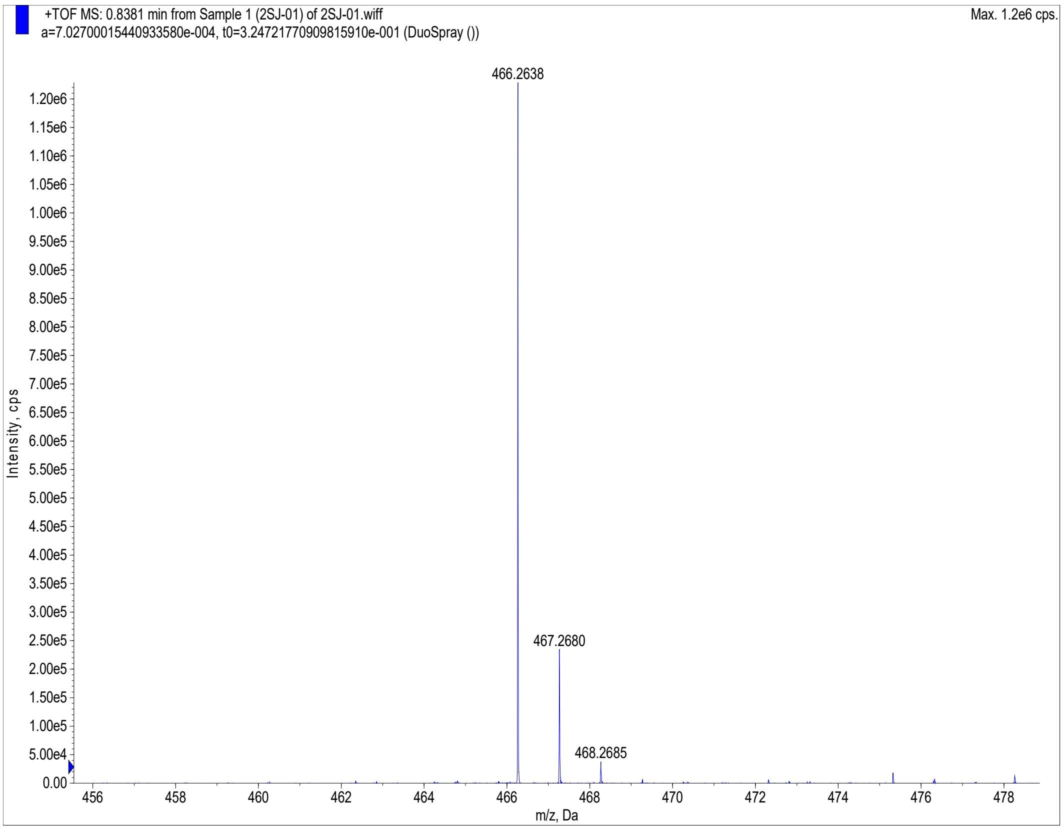

Figure S3. HR-MS spectrum of Xcn1.

HR-MS (m/z): $[M+H]^+$ calculated for $C_{22}H_{36}N_5O_6$, 466.2660; found, 466.2638.

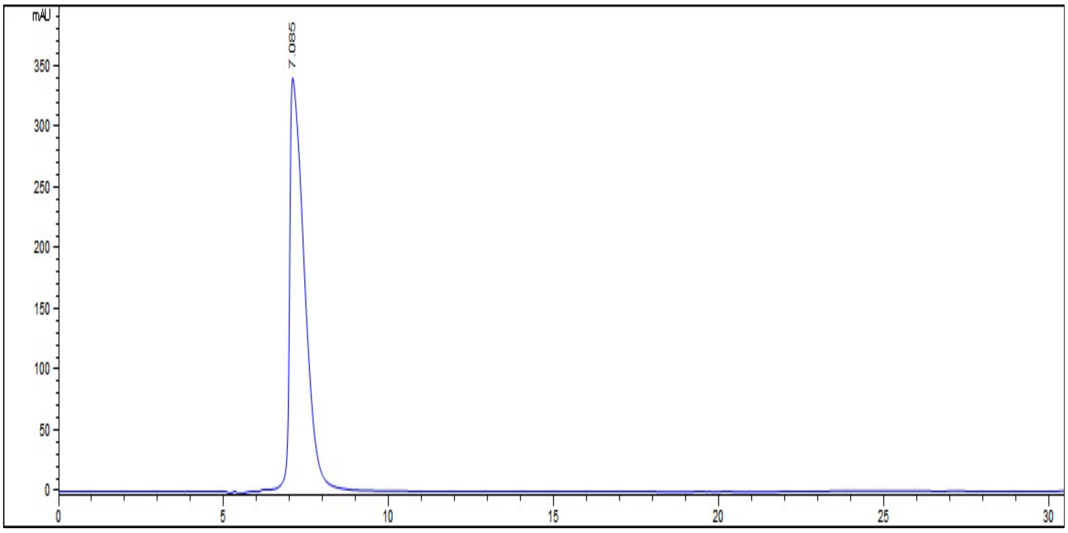

Figure S4. HPLC analysis of Xcn1.

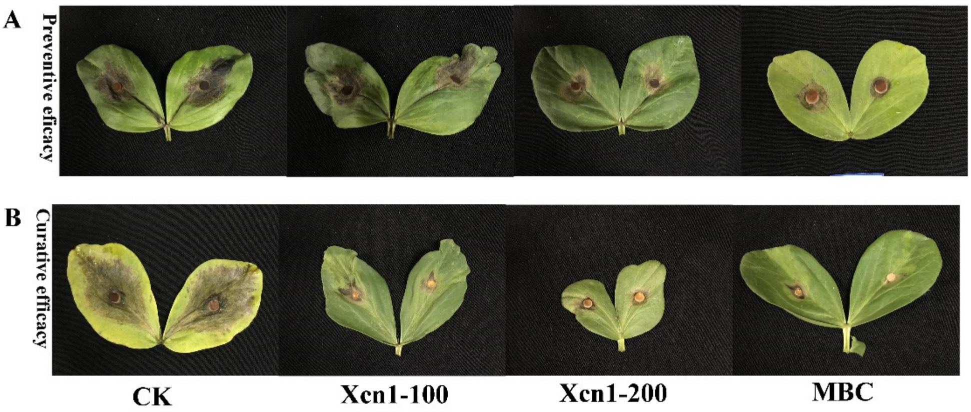

Figure S5. ***In vivo*** **antifungal activity of Xcn1 and carbendazim against** ***S. sclerotiorum*** **on broad bean. (A, C) Protective activity of Xcn1; (B, D) Curative activity of Xcn1. Preventive/curative efficacy: a sample solution was sprayed on broad bean leaves 12 h before/after inoculation with** ***S. sclerotiorum*****. Xcn1-100, 100 μg/mL of Xcn1; Xcn1-200, 200 μg/mL of Xcn1; MBC: 100 μg/mL of carbendazim.**

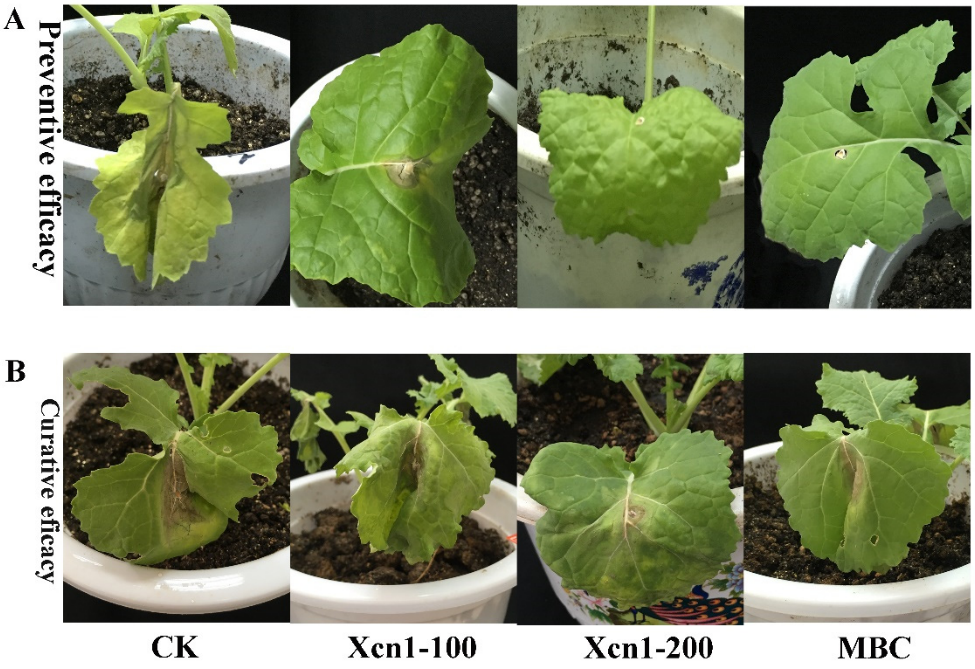

Figure S6. ***In vivo*** **antifungal activity of Xcn1 and carbendazim against** ***S. sclerotiorum*** **on rape seedlings. (A, C) Protective activity of Xcn1; (B, D) Curative activity of Xcn1. Preventive/curative efficacy: a sample solution was sprayed on broad bean leaves 12 h before/after inoculation with** ***S. sclerotiorum*****. Xcn1-100, 100 μg/mL of Xcn1; Xcn1-200, 200 μg/mL of Xcn1; MBC: 100 μg/mL of carbendazim.**

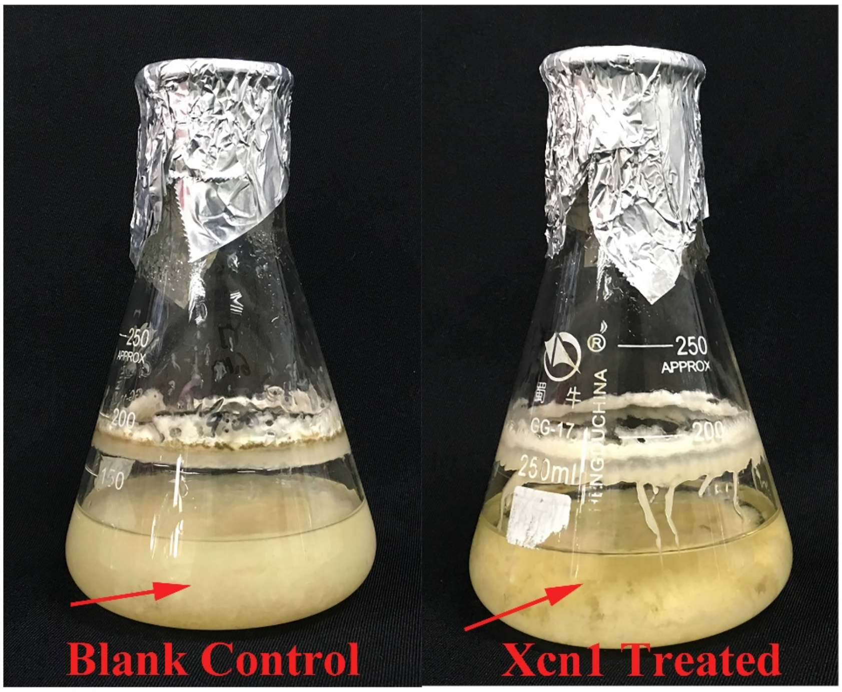

Figure S7. The appearance of the *S. sclerotiorum* culture treated with Xcn1 (3.0 μg/mL) or not (CK) for 7 days.

References:

Huang WR, Zhu CX, Yang XF, Yang HW, Xu HZ, Xie YY and Jian H (2005) Isolation and structural identification of main component CB6-1 produced by *Xenorhabdus nematophilus var. pekingensis*. Chinese Journal of Antibiotics 30(9):513-515

Mcinerney BV, Taylor WC, Lacey MJ, Akhurst RJ and Gregson RP (1991) Biologically active metabolites from *Xenorhabdus* spp., Part 2. Benzopyran-1-one derivatives with gastroprotective activity. Journal of Natural Products 54(3):785-795
